# Quantitative evaluation of aerosol generation during manual facemask ventilation

**DOI:** 10.1111/anae.15599

**Published:** 2021-10-26

**Authors:** A. J. Shrimpton, J. M. Brown, F. K. A. Gregson, T. M. Cook, D. A. Scott, F. McGain, R. S. Humphries, R. S. Dhillon, J. P. Reid, F. Hamilton, B. R. Bzdek, A. E. Pickering, the AERATOR study group, C White, J Murray, D Arnold, G Nava, N Maskell, J Dodd, E Moran, J Keller, M Gormley, S Sheikh, A Davidson

**Affiliations:** ^1^ Anaesthesia, Pain and Critical Care Sciences, School of Physiology, Pharmacology and Neuroscience University of Bristol Bristol UK; ^2^ Department of Anaesthesia and Intensive Care Medicine North Bristol NHS Trust Bristol UK; ^3^ School of Chemistry University of Bristol Bristol UK; ^4^ Department of Anaesthesia and Intensive Care Medicine Royal United Hospital NHS Trust Bath UK; ^5^ Department of Critical Care University of Melbourne; St. Vincent's Hospital Melbourne Australia; ^6^ Western Health Footscray Victoria Australia; ^7^ Climate Science Centre CSIRO Oceans and Atmosphere Aspendale Victoria Australia; ^8^ Department of Neurosurgery St Vincent's Hospital Melbourne Fitzroy Victoria Australia; ^9^ Department of Population Health Sciences University of Bristol Bristol UK

**Keywords:** aerosol‐generating procedure, COVID‐19, facemask ventilation, manual ventilation, SARS‐CoV‐2

## Abstract

Manual facemask ventilation, a core component of elective and emergency airway management, is classified as an aerosol‐generating procedure. This designation is based on one epidemiological study suggesting an association between facemask ventilation and transmission during the SARS‐CoV‐1 outbreak in 2003. There is no direct evidence to indicate whether facemask ventilation is a high‐risk procedure for aerosol generation. We conducted aerosol monitoring during routine facemask ventilation and facemask ventilation with an intentionally generated leak in anaesthetised patients. Recordings were made in ultraclean operating theatres and compared against the aerosol generated by tidal breathing and cough manoeuvres. Respiratory aerosol from tidal breathing in 11 patients was reliably detected above the very low background particle concentrations with median [IQR (range)] particle counts of 191 (77–486 [4–1313]) and 2 (1–5 [0–13]) particles.l^‐1^, respectively, p = 0.002. The median (IQR [range]) aerosol concentration detected during facemask ventilation without a leak (3 (0–9 [0–43]) particles.l^‐1^) and with an intentional leak (11 (7–26 [1–62]) particles.l^‐1^) was 64‐fold (p = 0.001) and 17‐fold (p = 0.002) lower than that of tidal breathing, respectively. Median (IQR [range]) peak particle concentration during facemask ventilation both without a leak (60 (0–60 [0–120]) particles.l^‐1^) and with a leak (120 (60–180 [60–480]) particles.l^‐1^) were 20‐fold (p = 0.002) and 10‐fold (0.001) lower than a cough (1260 (800–3242 [100–3682]) particles.l^‐1^), respectively. This study demonstrates that facemask ventilation, even when performed with an intentional leak, does not generate high levels of bioaerosol. On the basis of this evidence, we argue facemask ventilation should not be considered an aerosol‐generating procedure.

## Introduction

The COVID‐19 pandemic continues to place unprecedented demands on healthcare globally. The use of airborne personal protective equipment (PPE) has been reserved largely for healthcare workers undertaking medical procedures deemed to be aerosol generating [1, 2, 3]. These procedures are presumed to generate as much or higher levels of bioaerosols from the respiratory tract than coughing and consequently carry an increased risk of viral transmission. The evidence for these putative ‘aerosol‐generating procedures’ is predominantly epidemiological and from the time of the SARS‐CoV‐1 epidemic in 2003 [4, 5]. Several recent studies have questioned whether these medical procedures should be classified as ‘aerosol generating’ following quantitation of the aerosol produced during these activities [6, 7, 8].

Facemask ventilation is a core airway intervention and listed by the World Health Organization (WHO) as “*aerosol generating*” [4, 5]. The epidemiological evidence for this designation is from a single study that reported an increased risk of SARS‐CoV‐1 infection from facemask ventilation before tracheal intubation [9]. This risk was determined following interviews with 26 healthcare workers approximately 4 months after contracting SARS‐CoV‐1 to identify their clinical activity during the period 24 h before and 4 h following the performance of tracheal intubation. The authors reported a pooled increased risk of infection after being in the room of a SARS‐CoV‐1‐positive patient during tracheal intubation, where facemask ventilation had been performed (OR 2.8, 95%CI 1.3–6.4) [4, 9]. Twenty‐two of the 26 healthcare workers were infected by one patient and performing an electrocardiogram was associated with an even higher risk of SARS‐CoV‐1 infection (OR 3.5, 95%CI 1.6–7.9).

No study to date has quantified specifically the aerosol generated during facemask ventilation. Two recent clinical studies, performed in operating theatres, demonstrated relatively little aerosol generation for laryngoscopy and tracheal intubation [6, 10]. The analysis of the phase of facemask ventilation of the anaesthetised patient, before tracheal intubation, demonstrated conflicting results. Brown et al. reported that facemask ventilation was not aerosol‐generating [6]. In contrast, Dhillon et al. recorded an increased particle concentration above background during a period including facemask ventilation [10]. Resolution of these different findings is of crucial importance, as facemask ventilation is a key component of elective and emergency airway management. We therefore co‐developed an experimental protocol to test specifically whether facemask ventilation is a high‐risk procedure for aerosol generation. To assess the relative risk, we measured aerosol generation during facemask ventilation and compared this against tidal breathing and volitional coughs, with patients as their own controls.

## Methods

The study protocol was approved by the Greater Manchester Research Ethics Committee as part of the AERATOR study. The methods for aerosol measurement have been described previously [6]. In brief, a prospective environmental monitoring study was conducted in operating theatres in a UK hospital (Southmead Hospital, North Bristol NHS Trust). All recordings were made within operating theatres with an ultraclean ventilation system (EXFLOW 32, Howorth Air Technology, Farnworth, UK) placed in standby mode [11, 12]. This provides an environment with: very low background aerosol concentrations; an air change rate of 25 changes.h^‐1^ (in line with most other operating theatres in the UK); an air velocity of 0.25 m.s^‐1^ at 1 m above the ground; an air temperature of 20˚C; and relative humidity of 40–60%. An optical particle sizer (TSI Incorporated, model 3330, Shoreview, NM, USA) was used to record particle size, concentration and mass (within size range 300 nm–10 µm in diameter) at a sampling rate of 1 Hz. A 3D‐printed funnel was formed of polylactic acid on a RAISE3D Pro2 Printer, (3DGBIRE, Chorley, UK) with 90 mm height, 10 mm exit port and maximum diameter of 150 mm. This sampling funnel was connected to the optical particle sizer by a 1.25 m length of conductive silicone tubing of 4.8 mm internal diameter. All consented participants were aged over 18 y, ASA physical status 1 or 2, undergoing routine elective surgery requiring neuromuscular blockade before tracheal intubation and with a negative COVID‐19 polymerase chain reaction test in the previous 72 h. Patients with symptomatic gastro‐oesophageal reflux, a potential or known difficult airway or BMI ≥ 40 kg.m^‐2^ were not studied.

The clinical team undertook their normal practice during airway management with the single exception of intentionally generating a leak from the facemask during ventilation attempts after the induction of anaesthesia. The researchers were not involved in the delivery of peri‐operative care. All staff in the operating theatre wore airborne personal protective equipment including FFP3‐type masks. All participants were supine with head positioning as per the preference of the anaesthetist. An initial period of aerosol sampling was recorded with the patient awake which comprised of 60 s tidal breathing followed by three volitional coughs, spaced at 30 s intervals. Sampling was performed with the funnel 20 cm directly above the mouth of the patient. A piece of sampling tubing was cut to 20 cm and used to guide funnel positioning. The sampling funnel was then directed away from the patient to record background aerosol concentration in the operating theatre while the anaesthetist prepared the patient for induction of anaesthesia.

All patients were pre‐oxygenated with an F_I_O_2_ of 1.0. Induction of anaesthesia was performed with titrated intravenous propofol and opioid, followed by rocuronium at a dose of 0.4–0.6 mg.kg^‐1^. Once the patient was unconcious, facemask ventilation was performed with a circle breathing circuit connected to an anaesthetic machine (Aisys CS^2^, GE Healthcare, Chicago, IL, USA) enabling the measurement of airway pressure and the volume of manual breaths delivered. To ensure standardisation, 60 s of manual facemask ventilation was performed with a tidal volume of 5–7 ml.kg^‐1^ with airway pressures < 20 cm H_2_O and a respiratory rate of 12–15 breaths.min^‐1^. Standard anaesthetic monitoring, including waveform capnography and pulse oximetry, was used throughout. No airway adjuncts were required.

If the patient was clinically stable with their lungs easy to ventilate, the anaesthetist relaxed their grip on the mask to create an intentional, audible airway leak at the patient–mask interface. The patient’s lungs were then ventilated with the intentional leak for a further 60 s. The fresh gas flow and pressure limiting valve were adjusted to ensure the bag refilled to allow manual ventilation during this period. Monitoring of peripheral oxygen saturation was undertaken to ensure it did not fall below 95%, which would trigger restoration of ventilation with a good seal. During facemask ventilation with an intentional leak, the sampling funnel was positioned towards the side of the facemask towards the leak, maintaining a 20 cm distance from the mouth of the patient. The funnel was handheld to ensure it could be promptly removed in case of clinical need.

Airway management events were time‐stamped by the researcher, including: the period of tidal breathing; coughing; induction of anaesthesia; administration of neuromuscular blockade; start of recording for facemask ventilation with no leak; and start of facemask ventilation with a leak. Facemask ventilation analysis was commenced approximately 60 s after rocuronium had been administered. Aerosol sampling was continuous throughout the whole period from the induction of anaesthesia to tracheal intubation.

Based on previous work [6], the starting hypothesis was that facemask ventilation would produce no increase in aerosol above background. The sampling methodology was similar to previous work investigating aerosol production during gastro‐oesophageal endoscopy [11], where breathing was clearly distinguishable above background. Sample size calculations predicted 10 participants would ensure the study would be adequately powered to detect a difference of clinically important magnitude [13].

Data were processed in the TSI Aerosol Instrument Manager software, and analysed in Origin Pro (Originlab, Northampton, MA, USA) and Prism v9 (Graphpad, San Diego, CA, USA). The normality of data distribution was assessed using the Shapiro–Wilk test. Comparisons were made between aerosol measurements with parametric or non‐parametric statistical analyses as appropriate. The significance level was set at p < 0.05.

## Results

**Figure 1:**
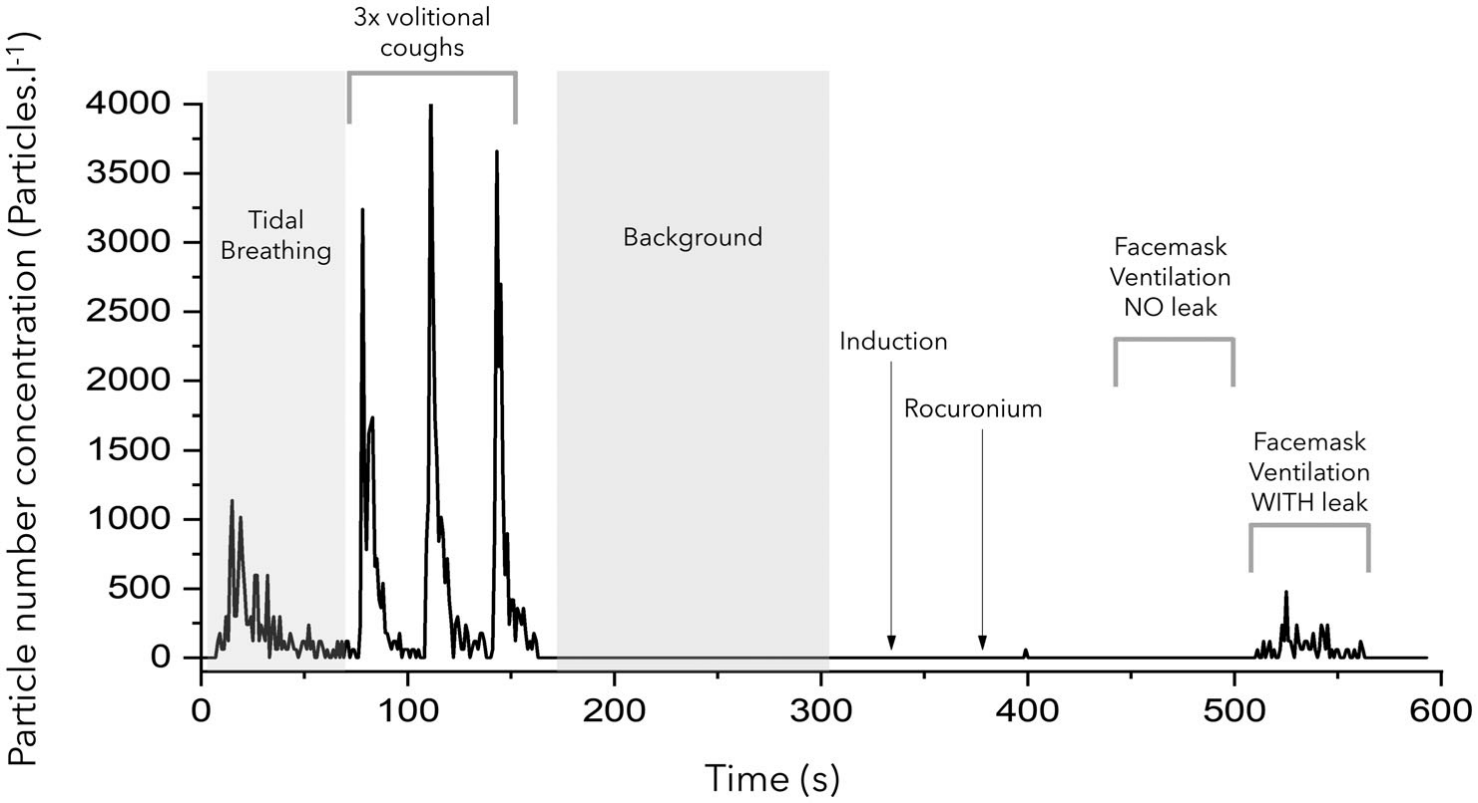
Aerosol concentration measured during the experimental protocol. This shows the concentration of particles detected during baseline respiratory manoeuvres (tidal breathing and voluntary coughs), background monitoring, facemask ventilation with no leak and facemask ventilation with a leak.

**Figure 2:**
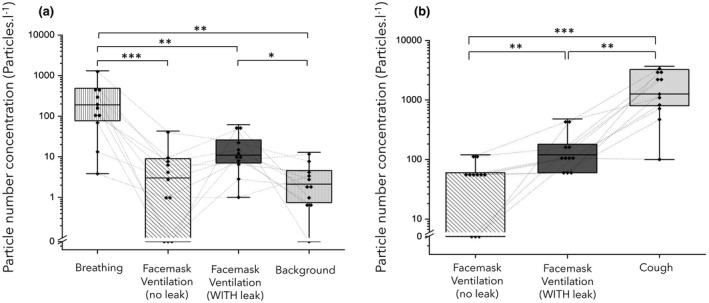
(a) Comparison of particle number concentrations detected during tidal breathing, facemask ventilation with/and without a leak and background levels. (b) Peak particle concentrations from facemask ventilation with/and without leak and cough. Boxes represent IQR, solid horizontal line represents median, Whiskers show range, [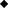] represents individual data points. Dotted lines link values for each participant. Wilcoxon matched pairs, *** p ≤ 0.001, ** p ≤ 0.01, * p ≤ 0.05.

Recordings were made during airway management for 11 patients undergoing elective surgery. There were six women and five men, mean (SD) age 60.0 (18.7) y and BMI 27.1 (5.0) kg.m^‐2^. The ultraclean ventilation system in the operating theatre environment produced a very low median (IQR [range]) background particle concentration of 2 (1–5 [0–13]) particles.l^‐1^. Spontaneous quiet tidal breathing was consistently detected above background levels (Fig. 1) with a particle concentration of 191 (77–486 [4–1313]) particles.l^‐1^, p = 0.002 (Fig. 2a).

The volitional coughs showed a peak aerosol concentration of 1260 (800–3242 [100–3682]) particles.l^‐1^. The analysis of the particle size distributions of these coughs demonstrated the distinctive ‘fingerprint’ of a cough observed in other studies with the majority of particles (86.5%) measuring < 1 µm in diameter [6, 11, 12, 14]. These coughs also had a characteristic temporal profile with a rapid increase in particles which decayed over the subsequent 15 s.

No airway difficulties were experienced and no request was made by the anaesthetists to remove the sampling funnel from the airway management zone. Peripheral oxygen saturation remained > 95% for all participants during facemask ventilation, including during the period of ventilation with an intentional leak. There were no coughs noted during facemask ventilation with or without a leak. The particle concentration detected during 60 s of facemask ventilation without a leak was 3 (0–9 [0–43]) particles.l^‐1^, which was no different to background (p = 0.43) and much lower than the concentration recorded during tidal breathing (p = 0.001) (Figs. 1 and 2a).

The particle concentration during facemask ventilation with a leak was 11 (7–26 [1–62]) particles.l^‐1^, which was approximately five‐fold higher than background (p = 0.019) but still much lower (17‐fold) than that seen during tidal breathing (p = 0.002). The analysis of the difference in particle concentration between facemask ventilation with and without a leak showed no statistically significant difference (p = 0.074) (Fig. 2a).

The peak particle concentration recorded during the periods of facemask ventilation without a leak was 60 particles.l^‐1^ (0–60 [0–120]) as compared with 120 particles.l^‐1^ (60–180 [60–480]) when there was a leak, which is 20‐fold (p = 0.002) and 10‐fold (p = 0.001) lower, respectively, than the particle count detected during a cough (Fig. 2b).

## Discussion

This study demonstrates that facemask ventilation in anaesthetised patients, even with a leak, generates less aerosol than tidal breathing and far less aerosol than a cough. This supports the findings from our previous study which included periods of facemask ventilation as part of the intubation sequence [6]. We found no evidence that the procedure of facemask ventilation in these circumstances generates high aerosol concentrations and therefore it should not be classified as an aerosol‐generating procedure [1, 15]. This has implications in a wide range of settings including during routine anaesthetic airway management. The avoidance of facemask ventilation before tracheal intubation or supraglottic airway insertion, due to concerns around aerosol generation, is not supported by this new evidence and likely serves only to increase the risk of encountering difficulties in airway management.

We have used tidal breathing and cough from participants to enable within‐subject comparison and relative risk estimation for facemask ventilation. Inter‐patient variation was considerable and ranged up to 50‐fold for tidal breathing and 36‐fold during coughing. This is in keeping with previous studies performed by the AERATOR group and others [6, 8, 11, 12, 16]. Therefore, using each participant as their own reference increases the power to generate meaningful comparisons from a relatively small sample. We have also modified our aerosol sampling position to move from 0.5 m to 0.2 m so as to be closer to the mouth of the patient. This has increased our ability to detect the emitted aerosol from source and has increased the concentration of particles recorded with tidal breathing and other respiratory activities. There was a 48‐fold increase in particle concentration detected during tidal breathing when recorded at this closer position (191 vs. 4 particles.l^‐1^) compared with our previous study of supraglottic airway devices performed in the same environment [12]. The higher measured particle concentration closer to the mouth are likely due to decreased particle dispersion and the capture of particles with low momentum in the smaller size range when sampling at 0.2 m compared with 0.5 m. Despite sampling closer to the source, it is possible some aerosol was not detected during facemask ventilation. However, as the concentration detected was far lower than that produced by tidal breathing, we can infer the relative risk of aerosol generation by facemask ventilation is very low.

The low concentration of aerosol detected during facemask ventilation with an intentional leak is also reassuring given that this represents a worst‐case scenario. The particles detected during facemask ventilation with leak likely represent respiratory aerosol originating from the lungs and upper airways during continued positive pressure ventilation rather than from turbulent airflow over the face. This is supported by the fact that these particles were predominantly small – which is consistent with respiratory origin where the smallest particles are thought to be generated [17]. We emphasise, however, that this concentration of aerosol was far lower than the patient would generate if conscious and breathing at rest. We can extend this conclusion further to state that a well‐fitting facemask with a good seal reduces emitted aerosol concentration to the point where it is indistinguishable from the near‐zero aerosol background, and we have previously demonstrated a well‐fitting facemask can prevent bioaerosol leak from a cough [12] by keeping respiratory aerosols within the breathing circuit. This is entirely predictable as the mask forms a physical barrier to aerosol spread.

A limitation of our study is that we intentionally studied a period of facemask ventilation after neuromuscular blockade to focus on the aerosol generation associated with the specific procedure rather than any paroxysmal respiratory event like coughing. However, aerosol sampling was conducted continuously throughout the induction of anaesthesia and the period of facemask ventilation performed immediately before the formally analysed period (i.e. before neuromuscular blockade) did not show increased aerosol concentrations above background (Fig. 1). Previous work performed by our group quantified aerosol generation during facemask ventilation in anaesthetised patients without neuromuscular blockade, which again did not demonstrate aerosol generation [12]. We are confident that our conclusions may be generalisable to the unparalysed patient.

Very low background particle counts, accurate time‐stamping and a high detector sampling rate are essential for accurate detection and attribution of aerosol from respiratory events and medical procedures. During the conduct of this study, we did not detect any non‐attributable spikes of aerosol. We have previously identified a variety of materials present in an operating theatre capable of generating high levels of (non‐respiratory) airborne particles, including: patient bedding; gauze; swabs; tube‐ties; throat packs; surgical scrubs; and incontinence pads [12]. The study by Dhillon et al., which reported episodes of increased aerosol during the period of facemask ventilation [10], was not performed in an operating theatre with an ultraclean ventilation system, making precise source attribution more challenging. There may also have been other procedures conducted during the induction sequence that could have generated aerosol, such as airway suctioning (currently listed as an aerosol‐generating procedure) which also requires further exploration.

In summary, this study demonstrates facemask ventilation, even when performed with an intentional leak, does not generate high levels of bioaerosol. Both tidal breathing and a volitional cough generate many‐fold more aerosol than facemask ventilation. On this basis, we believe facemask ventilation should not be considered an aerosol generating procedure. Accumulating evidence demonstrates many procedures currently defined as aerosol generating are not intrinsically high risk for generating aerosol, and that natural patient respiratory events often generate far higher levels [6, 7]. Furthermore, some of those procedures that generate aerosol, such as gastroesophageal endoscopy, only do so when the patient coughs [11]. The emerging evidence from quantitative clinical aerosol studies is yet to be incorporated into clinical guidance for aerosol generating procedures and we believe this needs urgent re‐assessment. Declassification of some of these anaesthesia‐related procedures as aerosol generating would seem appropriate due to their lack of aerosol generation. Our findings also raise the broader question of whether the term ‘aerosol generating procedure’ is still a useful concept for anaesthetic airway management practice in the prevention of SARS‐CoV‐2 or other airborne pathogens [18].

## Appendix 1

The Aerosolisation and transmission of SARS‐CoV‐2 in healthcare settings (AERATOR) study group are also authors of this study: C. White, J. Murray, D. Arnold, G. Nava, N. Maskell, J. Dodd, E. Moran (Southmead Hospital, North Bristol NHS Trust); J. Keller, M. Gormley (University Hospitals Bristol and Weston NHS Trust); S. Sheikh, A. Davidson (University of Bristol).
